# Characterization of the complete chloroplast genome of *Impatiens alpicola* (Balsaminaceae: Impatiens), a rare and endemic Chinese flowering plant

**DOI:** 10.1080/23802359.2019.1678418

**Published:** 2019-10-18

**Authors:** Qien Li, Xianjia Li, Maben Zhuo, Rangzhong Qieyang, Duojie Dongzhi, Xiao Guo

**Affiliations:** a Tibetan Medicine Research Center of Qinghai University, Qinghai University Tibetan Medical College, Xining, Qinghai, People’s Republic of China;; b State Key Laboratory of Tibetan Medicine Research and Development, Qinghai Tibetan Medicine Research Institute, Xining, Qinghai, People’s Republic of China

**Keywords:** Complete chloroplast genome, *Impatiens alpicola*, genome assembly, phylogeny

## Abstract

*Impatiens alpicola* is a newly recorded rare and endemic flowering plant in China, which has been regarded as threatened due to its narrow distribution and human activity. In this study, its complete chloroplast genome was assembled from the whole genome Illumina sequencing data. The circular genome was 151,366 bp long, containing a large single copy (LSC) region of 82,245 bp and a small single copy (SSC) region of 17,705 bp, which were separated by a pair of 25,708 bp inverted repeat (IR) regions. It encoded a total of 128 genes, including 76 protein-coding genes, 44 tRNA genes and eight rRNA genes. The most of gene species occurred as a single copy, while 18 gene species occurred in double copies. The overall A + T content was 63.1%, while the corresponding values of the LSC, SSC and IR regions were 65.4, 70.6, and 56.9%, respectively. Phylogenetic analysis indicated that *I*. *alpicola* was relatively close to another species (*I. piufanensis*) belonging to the same genus.

The genus *Impatiens* L., a member of the family Balsaminaceae, is one of the most species-rich genera of angiosperms, with over 1000 species distributed primarily in the Old World tropics and subtropics (Fischer 2004). Due to the zygomorphic flowers with tremendous diversity in corolla colour and morphology (Yuan et al. 2004), some species in this genus are of interest to horticulturalists (Yu et al. 2015). *Impatiens alpicola* Y. L. Chen & Y. Q. Lu is an annual herb and reported as a newly recorded species in this genus, with extremely limited distribution and unique habitat to Sichuan in China (Chen and Lu 1990; Chen et al. 2007). At the same time, this species has become threatened or their populations have declined dramatically due to its narrow distribution and human activity. In addition, Yu et al. (2015) investigated the phylogenetic relationships within the genus *Impatiens* using plastid and nuclear ITS variations. However, *I*. *alpicola* was not included in their study, the problem of its taxonomical phylogenetical position is still doubtful. In the present study, the complete chloroplast genome sequence of *I*. *alpicola* is reported for contributing to conservation of this species, and providing significant information for its phylogenetic placement.

Genomic DNA was extracted from fresh leaves of an individual of *I*. *alpicola* collected from the Emei Mountain in Sichuan province (103°18′33″E, 29°33′40″N; the specimen was deposited at Qinghai University; accession number: LQE-2017-089). The whole-genome sequencing was conducted on an Illumina Hiseq X Ten platform. The resultant clean reads were then assembled into complete chloroplast genome with the programme Velvet (Zerbino and Birney 2008), with *I*. *piufanensis* (GenBank: NC037401.1) as the starting reference. The complete chloroplast genome was annotated using Geneious (Kearse et al. 2012) and then submitted to GenBank (accession no. MK937571).

The chloroplast sequence of the *I*. *alpicola* was 151,366 bp. It possessed a typical quadripartite structure with two identical copies of a large inverted repeat separated by large and small single copy regions. The large single copy region (LSC) in *I. alpicola* was 82,245 bp and the small single copy region (SSC) was 17,705 bp, which were separated by a pair of 25,708 bp inverted repeat regions (IRs). The circular genome contained 128 genes, including 76 protein-coding genes (69 PCG species), eight ribosomal RNA genes (4 rRNA species) and 44 tRNA genes (31 tRNA species). The most of gene species occurred in a single copy, while 18 gene species occurred in double copies, including four rRNA species (4.5S, 5S, 16S, and 23S rRNA), 7 tRNA species and 7 PCG species. The overall A + T content of the circular genome was 63.1%, while the corresponding values of the LSC, SSC, and IR regions were 65.4, 70.6, and 56.9%, respectively.

A neighbour-joining tree (Figure 1) was reconstructed based on the complete chloroplast genome sequences of *I*. *alpicola* and two species from the family Balsaminaceae as well as other 18 species from the order Ericales, using the programme MEGA6 (Tamura et al. 2013). The phylogenetic analysis supported the traditional taxonomy of the order Ericales at the family level, *I*. *alpicola* was found to be relatively closely related to another species (*I*. *piufanensis*) from the same genus, and these two species were then clustered into a monophyletic clade with one specie (*Hydrocera triflora*) from the other genus of Balsaminaceae.

**Figure 1.**
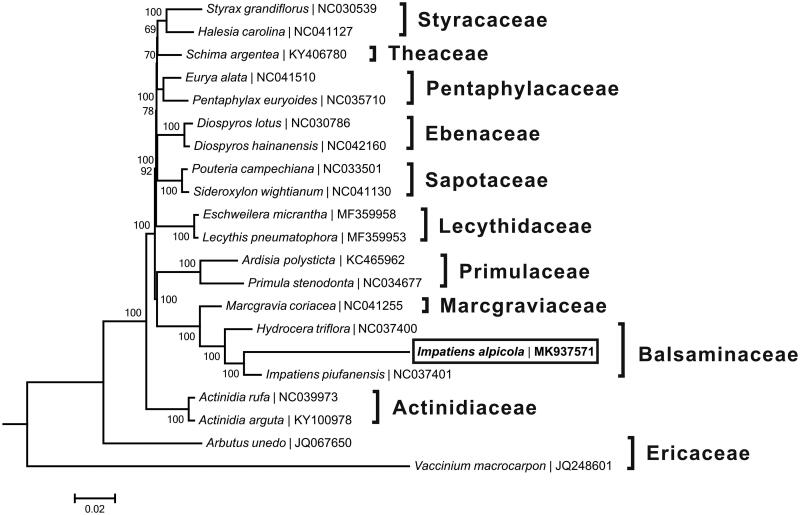
Neighbour-joining tree based on the complete chloroplast genome sequences of *Impatiens alpicola* and related taxa within the order Ericales. The numbers on the branches are bootstrap values. The accession number of GenBank for each species is listed in figure.
